# Understanding Hypoactive Sexual Desire Disorder (HSDD) in Women: Etiology, Diagnosis, and Treatment

**DOI:** 10.7759/cureus.49690

**Published:** 2023-11-30

**Authors:** Vaishnavi Ronghe, Krutika Pannase, Kavita P Gomase, Manjusha G Mahakalkar

**Affiliations:** 1 Obstetrics and Gynaecology, Smt. Radhikabai Meghe Memorial College of Nursing, Datta Meghe Institute of Higher Education and Research, Wardha, IND; 2 Obstetrics and Gynaecology Nursing, Smt. Radhikabai Meghe Memorial College of Nursing, Datta Meghe Institute of Higher Education and Research, Wardha, IND

**Keywords:** personalized interventions, sociocultural impact, treatment approaches, etiology and diagnosis, women's sexual health, hypoactive sexual desire disorder

## Abstract

Hypoactive Sexual Desire Disorder (HSDD) is a complex and multifaceted condition that significantly impacts the sexual well-being and overall quality of life of women. This comprehensive review aims to provide a holistic understanding of HSDD by exploring its etiology, diagnostic criteria, treatment approaches, and broader societal implications. The review delves into the intricate interplay of biological factors, including hormonal changes and neurotransmitter imbalances, that contribute to HSDD. Psychological factors, such as relationship issues, body image, and stress, are examined with sociocultural factors like societal norms, cultural influences, and media portrayals of sexuality. Diagnostic criteria and assessment methods, including The Diagnostic and Statistical Manual of Mental Disorders, Fifth Edition criteria, and self-report questionnaires, are explored to facilitate accurate identification of HSDD and differentiation from other sexual disorders. The impact of HSDD on women's quality of life and relationships is examined, highlighting the emotional strain and interpersonal challenges associated with the disorder. Societal and personal consequences of untreated HSDD underscore the need for increased awareness and support. Treatment approaches encompass non-pharmacological interventions such as cognitive-behavioral therapy, sex therapy, and couples therapy and pharmacological interventions like hormone therapy and selective serotonin reuptake inhibitors. Novel treatments like bremelanotide, flibanserin, and integrative strategies combining psychotherapy and lifestyle changes are discussed. Challenges and controversies surrounding HSDD, including the lack of consensus on diagnostic criteria, debates about the medicalization of sexuality, ethical concerns regarding pharmaceutical interventions, and cultural considerations, are addressed. Future directions in research, including advances in neurobiological understanding, personalized medicine, long-term treatment studies, and destigmatization initiatives, offer promising pathways for enhancing the management of HSDD.

## Introduction and background

Hypoactive Sexual Desire Disorder (HSDD) is a complex and multifaceted condition that significantly impacts the sexual well-being and overall quality of life of affected women. It is characterized by a persistent and distressing lack of interest in sexual activities, leading to personal and interpersonal difficulties [1,2]. Understanding and addressing HSDD in women is paramount due to its profound impact on personal relationships, self-esteem, and overall well-being. Intimate relationships often rely on a healthy and fulfilling sexual connection, and the absence of such desire can lead to emotional and psychological strain. Additionally, the stigmatization of sexual disorders underscores the significance of raising awareness and providing effective treatments [3].

This review offers a comprehensive and contemporary analysis of HSDD, specifically within the context of women. This review seeks to achieve several specific goals. First, it aims to distill and synthesize the most current research findings, theoretical perspectives, and clinical insights related to HSDD in women, drawing from various fields, including psychology, neurobiology, endocrinology, and sociology. Second, the review explores the intricate web of factors contributing to the development of HSDD in women. It seeks to unravel the complex etiological tapestry underlying the disorder's onset and persistence by delving into biological, psychological, and sociocultural influences. Moving beyond exploration, the review critically evaluates the diagnostic challenges associated with HSDD. This involves an examination of the existing diagnostic criteria and an exploration of the nuanced difficulties that can arise when attempting to accurately diagnose the disorder, particularly in the presence of potential overlap with other sexual dysfunctions.

## Review

Etiology of HSDD

*Biological* *Factors*

HSDD is a condition characterized by a deficiency in sexual desire, with hormonal imbalances playing a significant role in its development. The physiology of sexual desire involves complex interactions among multiple hormones and neurotransmitters. HSDD is likely a result of an overall reduction in sexual excitation signals, an increase in sexual inhibition signals, or a combination of both [4]. Testosterone, a key player in this scenario, is crucial for initiating sexual activities and fostering sexual desire and behavior. It also plays an essential role in regulating clitoral and vaginal physiology, facilitating genital lubrication, sensation, and engorgement [5]. Low levels of estrogen, on the other hand, are associated with dyspareunia and changes in vulvovaginal mucosa. These factors contribute to diminished sexual desire in affected women. Estrogen replacement therapy has been employed to boost sexual desire in postmenopausal women, with peripheral effects on addressing vaginal atrophy and dyspareunia, potentially reducing sexual pain and increasing desire [4].

While not explicitly addressed in the search results, progesterone is another hormone implicated in sexual desire. Progesterone receptors located in the hypothalamus, a key player in sexual desire regulation, suggest its potential role in modulating excitatory and inhibitory pathways in the brain, thereby impacting sexual desire [5]. The intricate web of neurotransmitters and peptides, such as serotonin, dopamine, and norepinephrine, further contributes to the physiology of sexual desire. HSDD may be linked to overactive inhibitory circuits in reward pathways, characterized by excessive serotonin activity and diminished dopamine activity [5]. Hormonal imbalances, encompassing changes in estrogen and progesterone levels, can result in shifts in sexual desire among women with HSDD. The exact mechanisms involve the intricate interplay among various hormones, neurotransmitters, and peptides, ultimately affecting the delicate balance between sexual excitation and inhibition signals in the brain [4,5].

*Psychological* *Factors*

Relationship issues can significantly impact sexual desire in women. Discord, lack of emotional intimacy, and communication difficulties within a relationship can lead to reduced sexual interest. Conversely, a positive and supportive relationship often correlates with higher levels of sexual desire [6].

Body image and self-esteem also play a critical role in HSDD. Negative body image perceptions and low self-esteem can create barriers to feeling desirable and sexually confident, dampening sexual motivation [7]. Stress and mood disorders, such as depression and anxiety, can substantially affect sexual desire. The psychological burden of these conditions can lead to a decreased focus on sexual experiences and diminished interest in sexual activities [8].

*Sociocultural* *Factors*

Societal norms and expectations surrounding female sexuality can exert substantial influence. Cultural factors that dictate how women should express their desires and behaviors may contribute to feelings of guilt or shame, suppressing authentic sexual expression [9].

Cultural influences on sexuality, including religious beliefs and traditional values, can shape individuals' attitudes toward their sexuality and impact the prioritization of sexual desire within relationships [10]. Media portrayal of sexuality can also contribute to HSDD by creating unrealistic expectations and standards. The prevalence of idealized and often unrealistic images of sexual encounters in media can lead to dissatisfaction with one's experiences, resulting in diminished sexual desire [11].

Diagnostic criteria and assessment

*Diagnostic and Statistical Manual of Mental Disorders, Fifth Edition* *Criteria for HSDD Diagnosis*

The Diagnostic and Statistical Manual of Mental Disorders, Fifth Edition, establishes standardized criteria for diagnosing HSDD. To meet these criteria, there must be a persistent lack or reduction of sexual desire over a substantial period, leading to distress or interpersonal difficulties. It is imperative to differentiate between general periods of low desire and the more enduring nature characteristic of HSDD [2].

Differentiating Between HSDD and Other Sexual Disorders

It is essential to carefully distinguish HSDD from other sexual disorders, such as erectile dysfunction in men or female arousal disorder. While these disorders may exhibit similarities, HSDD predominantly revolves around the absence or reduction of sexual desire rather than challenges related to physical arousal [12].

Diagnosing HSDD

Diagnosing HSDD can pose challenges due to various factors. Cultural and societal attitudes toward female sexuality may contribute to underreported symptoms, as women might be hesitant to discuss their low desire. Additionally, the subjective nature of desire and the absence of a universally defined "normal" level of sexual desire can complicate the diagnosis [3].

Clinical Interviews and Self-Report Questionnaires

Trained professionals play a pivotal role in the diagnostic process by conducting clinical interviews. Open and non-judgmental discussions can extract information about a patient's sexual history, relationship dynamics, and psychological well-being. Standardized self-report questionnaires, such as the Female Sexual Function Index or the Decreased Sexual Desire Screener, offer tools to quantify and assess sexual desire and associated distress [13].

Role of Medical and Sexual History Assessment

Obtaining a comprehensive medical history is critical, given that medical conditions (e.g., hormonal imbalances, chronic illnesses) and medications (e.g., antidepressants) may contribute to HSDD. A thorough sexual history assessment explores the onset and duration of the disorder, changes over time, and any triggering events or factors [14].

Prevalence and impact

Prevalence of HSDD in Different Age Groups

The prevalence of HSDD is higher in certain age groups. For example, in the US, the prevalence of HSDD was 8.9% in women aged 18-44 years, 12.3% in women aged 45-65 years, and 7.4% in women aged 65 years or older [13]. Surgically menopausal women were found to have a higher prevalence of HSDD compared to premenopausal or naturally menopausal women, regardless of their age at the time of the interview or when their ovaries were removed [14]. The prevalence of HSDD was found to be significantly higher among American women in the 30-39 age range compared with European women of the same age [13]. In Europe, the prevalence of HSDD ranged from 6% to 13%, while in the US, it ranged from 12% to 19% [12]. Postmenopausal women often experience lower levels of sexual desire, but no identifiable hormonal differences characterize premenopausal women with HSDD [15]. Factors such as race/ethnicity, educational level, body mass index, current smoking status, and current depression were also found to be associated with variations in the prevalence of low desire and HSDD [14].

Impact on Quality of Life Age Relationships

HSDD can profoundly impact the quality of life of affected women. Diminished sexual desire can lead to emotional distress, reduced self-esteem, and inadequacy. The strain may extend beyond the individual, affecting intimate relationships. Partners may experience frustration, confusion, and rejection due to the lack of sexual interest. This strain can lead to communication breakdowns and conflict within the relationship [16]. Furthermore, the emotional disconnect caused by HSDD can create a barrier to emotional intimacy, contributing to a cycle of reduced sexual desire and strained relationships. The emotional toll on both individuals can increase stress and potentially exacerbate existing psychological conditions.

Societal and Personal Consequences of Untreated HSDD

Untreated HSDD can have wide-ranging societal and personal consequences. On a personal level, the emotional distress and lowered self-esteem resulting from HSDD can negatively impact mental health and overall well-being. The lack of sexual fulfillment and the associated shame or guilt can erode self-confidence, affecting various aspects of a woman's life beyond her intimate relationships [17]. Societally, untreated HSDD can perpetuate harmful stigmas around female sexuality. It can reinforce unrealistic expectations of sexual desire and function, leading to misunderstanding and misrepresenting women's experiences. The silence surrounding sexual disorders can prevent women from seeking help, perpetuating the cycle of untreated HSDD and its associated negative consequences.

Treatment approaches

Addressing HSDD in women requires a multifaceted approach that combines non-pharmacological interventions, pharmacotherapy, novel treatments, and integrative strategies to alleviate symptoms and improve overall sexual well-being effectively.

Non-pharmacological Interventions

Cognitive-behavioral therapy: Cognitive-behavioral therapy (CBT) is a psychological approach that offers individuals a structured framework to identify and modify negative thought patterns and behaviors contributing to their experiences of HSDD. In the context of HSDD, CBT helps women recognize and challenge distorted beliefs about their bodies, sexuality, and self-worth. By working closely with a trained therapist, individuals can learn to reframe these perceptions, replacing them with more accurate and positive thoughts. This process fosters healthier attitudes toward their sexuality, helping to alleviate the psychological barriers that hinder sexual desire. CBT equips women with practical strategies to manage stress, address self-esteem issues, and develop coping mechanisms that positively impact their sexual well-being [18].

Sex therapy and counseling: Sex therapy and counseling provide a specialized and confidential space for individuals and couples to address various sexual concerns, including those related to HSDD. Trained therapists guide open discussions that explore an individual's or couple's sexual experiences, feelings, and challenges. This approach goes beyond the physical aspects of sexuality, delving into emotional and relational dynamics. Sex therapy focuses on enhancing intimacy and improving overall sexual satisfaction through communication skills, education, and strategies to overcome obstacles. It offers a supportive environment where individuals can learn techniques to enhance arousal, foster emotional connection, and navigate the complexities of sexual desire. For couples, sex therapy helps strengthen their bond, address misunderstandings, and collaboratively work towards shared sexual goals [19].

Couples therapy: Couples therapy is particularly relevant when HSDD affects both partners within a relationship. The disorder can lead to communication breakdowns, emotional distance, and unmet expectations related to sexual intimacy. Couples therapy addresses these challenges by facilitating productive discussions in a safe, non-judgmental space. Therapists help couples explore the emotional and relational aspects of HSDD, guiding them to express their feelings, concerns, and desires openly. By promoting effective communication, couples can better understand each other's perspectives, alleviate emotional disconnect, and collaboratively work towards solutions. The therapy aims to improve relationship dynamics, restore emotional intimacy, and enhance overall relational satisfaction. Ultimately, couples therapy equips partners with tools to navigate the impact of HSDD on their relationship, fostering a stronger and more resilient bond [20].

Pharmacological Interventions

Hormone therapy: Hormone therapy involves the administration of hormones, such as estrogen and progesterone, to address hormonal imbalances. This approach is particularly relevant for women experiencing HSDD due to hormonal changes, such as those occurring during menopause or due to certain medical conditions. Menopause, for example, is associated with decreased estrogen levels, leading to a decline in sexual desire and overall sexual function [21]. Hormone therapy aims to restore hormonal levels to a more balanced state. By replenishing estrogen, this treatment can alleviate some of the symptoms of HSDD, including reduced libido. It can also address other menopause-related symptoms, such as vaginal dryness and discomfort during intercourse, contributing to sexual difficulties [21].

However, hormone therapy is not without potential risks and considerations. There have been discussions about the benefits versus potential risks, including an increased risk of certain health conditions like breast cancer, blood clots, and cardiovascular issues. The decision to pursue hormone therapy should involve thoroughly evaluating an individual's medical history and risk factors and carefully considering the potential benefits and risks [22].

Selective serotonin reuptake inhibitors and other medications: Selective serotonin reuptake inhibitors (SSRIs), commonly used as antidepressants, have been explored as a potential pharmacological treatment for HSDD. These medications impact neurotransmitters in the brain, particularly serotonin, which affects mood regulation. Off-label use of SSRIs for HSDD is based on their potential to increase serotonin levels and, as a result, influence sexual desire [23].

However, using SSRIs and other medications for HSDD is a subject of ongoing debate and research. Their efficacy can vary widely among individuals, and not all women may experience a significant improvement in sexual desire. Additionally, these medications can be associated with side effects, such as changes in mood, gastrointestinal issues, and sexual side effects like decreased arousal or delayed orgasm [14]. Before considering SSRIs or other medications for HSDD, a comprehensive assessment by a healthcare professional is essential. Factors like overall health, medications taken, and potential interactions must be considered.

Novel and Emerging Treatments

Bremelanotide (Vyleesi) and Flibanserin (Addyi) are two FDA-approved medications that have been developed with the explicit goal of addressing HSDD in women. These medications represent distinct pharmacological approaches targeting the intricate factors contributing to HSDD [24].

Bremelanotide (Vyleesi): Bremelanotide operates as a melanocortin receptor agonist. By engaging with specific receptors in the brain's neural pathways associated with sexual desire and arousal, bremelanotide aims to stimulate and enhance the neural circuits that govern sexual motivation. This engagement with the brain's signaling mechanisms encourages increased sexual desire and a more positive emotional response to sexual stimuli. Bremelanotide is administered through a self-administered injection and is intended to be used as needed, allowing women to initiate the medication when they anticipate sexual activity [25].

Flibanserin (Addyi): Flibanserin operates as a serotonin receptor modulator through a different mechanism. It targets specific serotonin receptors in the brain, which regulate mood and emotions. By modulating serotonin receptor activity, flibanserin aims to rebalance neurochemical systems that might contribute to reduced sexual desire. It is important to note that flibanserin requires consistent, daily use. It is intended to take some time to influence neural pathways related to sexual motivation and satisfaction gradually [26].

Investigational drugs and therapies: As the field of HSDD research evolves, ongoing studies are exploring new avenues for pharmacological interventions. These investigations encompass a range of approaches that target various neural pathways, neurotransmitter systems, and hormonal interactions. The goal is to identify novel compounds or combinations of existing medications that can effectively address HSDD's complex etiology. These investigational drugs and therapies hold the potential to offer additional options for women who do not respond optimally to currently available treatments. As these potential interventions are being studied, it is crucial to rigorously evaluate their safety, efficacy, and potential side effects through well-designed clinical trials [27].

Integrative approaches

Combination of Psychotherapy and Medication

The combination of psychotherapy and medication represents a holistic and synergistic approach to addressing HSDD. By merging psychological interventions like CBT or sex therapy with pharmacological treatments, this approach simultaneously targets the psychological and neurobiological aspects of HSDD [18].

Psychotherapy, such as CBT or sex therapy, delves into the underlying psychological factors contributing to HSDD. Therapists work collaboratively with individuals to identify and challenge negative thought patterns, address relationship dynamics, and foster healthy sexual communication. CBT, for instance, helps individuals reframe distorted beliefs about their body image, self-esteem, and sexuality, thereby improving self-perception and body confidence. Sex therapy facilitates open discussions about sexual concerns, relationship issues, and intimacy barriers, promoting emotional connection and sexual satisfaction [28].

Complementing psychotherapy with medication addresses the potential neurochemical imbalances that may contribute to HSDD. Medications like bremelanotide or flibanserin aim to modulate neurotransmitter systems linked to sexual desire, offering a pharmacological means to augment libido. Integrating these medications can enhance the neurobiological aspects of desire, working in tandem with the psychological strategies of psychotherapy [29].

Lifestyle Changes

Incorporating healthy changes can significantly improve overall well-being and potentially revive sexual desire. Regular exercise, for instance, has been shown to enhance mood, reduce stress, and improve blood circulation - factors that can positively influence sexual function and desire. Physical activity can increase energy levels and promote a positive body image, fostering a healthier attitude towards one's body and sexual experiences [30]. Balanced nutrition is equally crucial. A diet rich in nutrients, vitamins, and minerals supports hormonal balance and general physiological functioning, impacting sexual health. Certain foods, such as those high in antioxidants and omega-3 fatty acids, are linked to improved blood flow and may contribute to sexual arousal [31].

Adequate sleep is another fundamental aspect of lifestyle changes. Sleep deprivation can lead to increased stress, hormonal imbalances, and reduced energy levels, negatively impacting sexual desire. Prioritizing restful sleep can enhance well-being and promote a more positive sexual experience. It is important to note that before embarking on significant lifestyle changes, consulting healthcare professionals is advised. They can provide personalized guidance based on individual health conditions, ensuring that modifications to exercise, diet, or sleep align with overall health goals. This proactive approach enhances the safety and effectiveness of lifestyle adjustments, emphasizing the importance of a collaborative effort between individuals and healthcare providers for comprehensive well-being [30,31].

Challenges and controversies

Lack of Consensus on Diagnostic Criteria

One of the significant challenges in the field of HSDD is the lack of a universally agreed-upon set of diagnostic criteria. The subjective nature of sexual desire and variations in cultural and individual norms make it difficult to establish clear-cut boundaries for diagnosing HSDD. This lack of consensus can lead to varying prevalence rates and hinder accurate assessment and treatment [2].

Debates Around Medicalization of Sexuality

There are ongoing debates surrounding the medicalization of sexuality, particularly in HSDD. Some argue that pathologizing variations in sexual desire can contribute to unnecessary medical interventions and undermine the natural diversity of human sexuality. Critics emphasize the importance of considering sociocultural factors and individual preferences in defining "normal" sexual desire [32].

Ethical Concerns Regarding Pharmaceutical Interventions

Introducing pharmaceutical interventions for HSDD, such as bremelanotide (Vyleesi) and flibanserin (Addyi), has sparked ethical debates. Questions arise about the appropriate role of medications in addressing complex psychological and relational issues. Concerns include potential overreliance on drugs and the risk of neglecting non-pharmacological interventions that may address underlying causes [24].

Cultural Considerations and Cross-Cultural Variations

Cultural norms and values significantly impact perceptions of sexual desire and behavior. What may be considered normal or problematic can vary widely across cultures. This raises questions about the applicability of Western diagnostic criteria to diverse cultural contexts and the potential for misinterpretation or misdiagnosis [33].

Future directions

Advances in Understanding HSDD Neurobiological Basis

Continued research into the neurobiological underpinnings of HSDD holds the potential to uncover new insights. Exploring brain pathways, neurotransmitter systems, and hormonal interactions can provide a deeper understanding of the mechanisms that drive diminished sexual desire. These insights may lead to targeted interventions that address specific neurobiological factors.

Personalized medicine approaches: Advancements in genetics and personalized medicine could pave the way for tailored treatments for HSDD. By identifying genetic markers or individual differences in hormone levels, neurotransmitter function, or other relevant factors, healthcare professionals could design personalized interventions that are more effective and have fewer side effects.

Long-term studies on treatment efficacy and safety: As newer treatments, such as bremelanotide and flibanserin, gain traction, conducting rigorous, long-term studies on their efficacy and safety is imperative. Gathering comprehensive data on treatment outcomes, potential side effects, and long-term impacts can guide clinical decision-making and provide a more nuanced understanding of these interventions' benefits and limitations.

Destigmatization and awareness campaigns: Efforts to destigmatize HSDD and other sexual disorders are crucial for encouraging open discussions and seeking help. Public awareness campaigns can provide accurate information about HSDD, challenge misconceptions, and promote empathy. By fostering a culture of understanding and support, individuals affected by HSDD can feel more empowered to seek assistance and share their experiences.

## Conclusions

In conclusion, the exploration of HSDD reveals a complex interplay of biological, psychological, and sociocultural factors contributing to its manifestation and impact on women's lives. The diverse array of factors, from hormonal changes and neurotransmitter imbalances to relationship dynamics and societal norms, underscores the need for a comprehensive understanding of HSDD. Accurate diagnosis, aided by standardized criteria and thorough assessments, is crucial for effective intervention. The far-reaching consequences of HSDD, encompassing emotional distress and strained relationships, emphasize the necessity of targeted treatments that address the disorder's individual and relational aspects. The evolving treatment landscape presents a range of options, from psychotherapy to pharmacological interventions, each catering to different dimensions of HSDD. Integrating psychotherapy with medication offers a synergistic approach, addressing psychological and neurobiological factors. Lifestyle changes emerge as a significant contributor, reflecting the interconnectedness of physical and sexual well-being. Challenges surrounding diagnosis, medicalization, and cultural variations underscore the complexity of HSDD and the importance of an inclusive and nuanced approach. The trajectory of HSDD research points toward a future characterized by advancements in neurobiological understanding, personalized interventions, and long-term treatment studies, offering hope for improved management. Moreover, the call for destigmatization and awareness campaigns signifies a societal shift toward open conversations, empathy, and support for women grappling with HSDD.
